# Elements of Materia Medica and Pharmacy

**Published:** 1842-04

**Authors:** 


Art. IX.—*Elements of Materia Medica and Pharmacy.* By O'Bryan Bellingham, m.d. &c. Edited by Arthur Mitchell, m.d. Part I. —*Dublin,* 1841. 8vo, pp. 302.

The volume before us contains sufficiently full and perspicuous information on all the matters which its title implies. The following is a general statement of the author's plan. First, we have a chemical analysis of the articles of the Materia Medica. The division adopted in regard to the elementary substances (which are, of course, first discussed), is that of non-metallic and metallic; after which, the metallic oxides, the acids, alkalies, and the salts, are successively treated of. Chapter v. is devoted to a consideration of the proximate principles of vegetables, to the modes of analysing and classifying these. Chapter vi. is divided into two parts, of which the first is occupied with the neutral vegetable principles, among which we may enumerate arabin, bassorin, cerasin, amidin, mucin, glutin; in the second part, the intermediate bodies are examined, such as the fixed oils, stearin, resins, alcohol, chlorophyllite. Then follow the vegetable acids and vegetable alkalies. Chapter vii. is taken up with an account of the general nature of pharmaceutical operations. An account, *in abstracto*, of the pharmaceutical preparations, occupies Chapter viii. Chapters ix. x. and xi. are respectively devoted to the *modus operandi* of medicines, to the circumstances which modify the general actions of medicines, and to the modes of administering and combining these; and Chapter xii. which concludes this volume and the first part of the work, gives the classification of the Materia Medica, first according to the natural-historical, and secondly, according to the therapeutic arrangement.

